# Is the C-C Motif Ligand 2–C-C Chemokine Receptor 2 Axis a Promising Target for Cancer Therapy and Diagnosis?

**DOI:** 10.3390/ijms21239328

**Published:** 2020-12-07

**Authors:** Hiroaki Iwamoto, Kouji Izumi, Atsushi Mizokami

**Affiliations:** Department of Integrative Cancer Therapy and Urology, Kanazawa University Graduate School of Medical Science, 13-1 Takara-machi, Kanazawa 920-8641, Ishikawa, Japan; hiroaki017@yahoo.co.jp (H.I.); mizokami@staff.kanazawa-u.ac.jp (A.M.)

**Keywords:** biomarker, CCL2, CCR2, chemokine, immunotherapy, cancer, tumor, progression, metastasis

## Abstract

C-C motif ligand 2 (CCL2) was originally reported as a chemical mediator attracting mononuclear cells to inflammatory tissue. Many studies have reported that CCL2 can directly activate cancer cells through a variety of mechanisms. CCL2 can also promote cancer progression indirectly through increasing the recruitment of tumor-associated macrophages into the tumor microenvironment. The role of CCL2 in cancer progression has gradually been understood, and various preclinical cancer models elucidate that CCL2 and its receptor C-C chemokine receptor 2 (CCR2) are attractive targets for intervention in cancer development. However, clinically available drugs that regulate the CCL2–CCR2 axis as anticancer agents are not available at this time. The complete elucidation of not only the oncological but also the physiological functions of the CCL2–CCR2 axis is required for achieving a satisfactory effect of the CCL2–CCR2 axis-targeted therapy.

## 1. Introduction

C-C motif ligand 2 (CCL2) was discovered first among CC chemokines in 1989 and helped to propel subsequent discoveries of other CC chemokines [1]. CCL2, also known as monocyte chemoattractant protein-1 (MCP-1), is composed of 76 amino acids and has a size of 13 kDa [2]. CCL2 is expressed in a wide range of cells, including endothelial cells, epithelial cells, myeloid cells, smooth muscle cells, and fibroblasts [3]. The biological function of CCL2 is mediated through its G protein-coupled receptor C-C chemokine receptor 2 (CCR2) [4]. Although CCR2 also binds to CCL7, CCL8, CCL12, and CCL13, CCL2 most potently activates a CCR2-mediated signaling pathway [5]. CCL2 has two specific high-affinity receptors, CCR2A and CCR2B, and activation of monocytes by CCL2 mediates monocyte infiltration into tissues in inflammatory diseases [6]. The number of studies on the role of CCL2 in the development of obesity, diabetes, cardiovascular diseases, insulitis, diabetic nephropathy, and diabetic retinopathy has increased exponentially over time [7,8,9]. In addition, studies on the role of CCL2 in cancer have been reported since 2000, and the role of CCL2 in the tumor microenvironment, immuno-oncology, and cancer cells has been the main topic of CCL2 research nowadays. Other chemokines, such as CCL18 and CCL20, have been reported to play important roles in the growth and migration of various cancers and have been shown to be promising as tumor markers and therapeutic targets, but have not yet reached clinical application [10,11]. Although the roles of CCL2 in cancer progression have been understood gradually, drugs modulating the CCL2–CCR2 axis as anticancer agents are clinically not available except for administration in clinical trials. Because of the multifunctional nature of CCL2, the blockade of the CCL2–CCR2 axis may cause unexpected effects on cancer patients. The potentials and limitations of CCL2–CCR2 axis targeted as cancer immunotherapy and a diagnostic tool are described in this review.

## 2. Role of the CCL2–CCR2 Axis in Cancer Cells

### 2.1. The Direct Effects of CCL2 on Cancer Cells

There is a number of studies suggesting that CCL2 can activate cancer cells through a variety of mechanisms. Initially, CCL2 was focused on and studied as a key molecule in the migration and proliferation of prostate cancer cells [12]. CCL2 directly stimulates prostate cancer cell proliferation and migration via activation of the phosphatidylinositol 3-kinase (PI3K)/ protein kinase B (Akt) signaling pathway and Rac GTPase, respectively [13]. CCL2 induces cancer cell migration and contributes to the early stages of metastasis by interacting with CCR2 expressed on cancer cells [14]. Cancer cells acquire a migratory and invasive phenotype that invades adjacent tissues by disrupting the extracellular matrix and migrates toward the blood and lymphatic vessels in an early stage of CCL2-induced metastasis [15,16]. CCL2-dependent Signal Transducers and Activator of Transcription 3 (STAT3) activation and the induction of epithelial-mesenchymal transition (EMT) pathways have been reported, and CCL2 modulates the expression of EMT-related proteins, matrix metalloproteinase (MMP) 2, MMP9, Vimentin, Snail, and E-cadherin in cancer cells [17,18,19,20,21,22,23,24,25,26,27]. Interruption of the CCL2–CCR2 axis can suppress EMT and prostate cancer cell migration and invasion, providing a critical mechanism linking CCL2 and cancer metastasis [19,22,23,24,27,28]. CCL2 may be a starting point for subsequent chemokine cascades inducing prostate cancer cell activation [29]. CCL2 contributes to the acquisition of resistance to taxane chemotherapies such as docetaxel and cabazitaxel in prostate cancer cells [30,31]. CCL2 also contributes to the acquisition of tamoxifen resistance in breast cancer cells [32].

Although a great number of the studies focusing on the function of CCL2 in cancer progression used prostate and breast cancer cells, more recent CCL2 studies have incorporated colorectal, lung, gynecological, gastric, and other types of cancer cells. Akt activation by phosphatidylinositol phosphate kinase gamma (PIPKIγ)-mediated STAT3 phosphorylation induces CCL2 expression in colorectal cancer cells and silencing PIPKIγ greatly decreases CCL2 expression at both the mRNA and protein levels, leading to reduced chemotaxis of cancer cells to macrophages [33]. In metastatic colorectal cancer liver metastatic model mice, administration of CCR2 inhibitor improves the chemotherapy response and prolongs overall survival of cancer-bearing mice [34]. Targeting CCR2 with its antagonist also suppresses viability, motility, and invasion by downregulating MMP-9 expression in non-small cell lung cancer cells [35]. The CCL2–CCR2 axis enhances interleukin (IL)-6-induced EMT by cooperatively activating STAT3–Twist signaling in non-small cell lung cancer cells [20]. Although the detailed mechanism is not clarified, CCL2 promotes invasion and adhesion of human ovarian cancer cells, and CCL2 blockade can enhance the cytotoxic effect of paclitaxel and carboplatin therapies in ovarian cancer [36,37]. CCL2 also contributes to the acquisition of resistance to docetaxel in lung cancer and resistance to cisplatin in gastric cancer [38,39].

### 2.2. The Effects of CCL2 on Cancer Cell via Tumor Microenvironment

Since CCL2 is also known by the alias monocyte chemoattractant protein-1 and activates macrophages, inactivation of cancer cells by the inhibition of the CCL2–CCR2 axis are partly presumed to be caused by the reduction of tumor-associated macrophages (TAMs) at the primary and metastatic site, inducing the destruction of the immunosuppressive tumor microenvironment and the susceptibility to the antitumor T lymphocyte response. TAMs play a major role in cancer progression by affecting diverse processes such as immunosuppression, angiogenesis, and tumor cell proliferation and metastasis during cancer progression [40,41,42]. It was reported that CCL2-positive prostate cancer tissues have more macrophage infiltration than CCL2-negative cancer tissues, CCL2 can also promote prostate cancer progression indirectly by increasing the recruitment of TAMs into the tumor microenvironment [28,43]. An increase in the number of TAMs generally shortens survival and increases the grade and stage of cancer. Specifically, they have been reported in gastric cancer, breast cancer, prostate cancer, classical Hodgkin’s lymphoma, kidney cancer, nasopharyngeal cancer, and glioma [44,45,46,47,48,49,50,51,52]. CCL2-induced chemokine cascade promotes breast cancer metastasis by enhancing retention of TAMs [53]. Radiotherapy leads to a significant increase in CCL2 production by pancreatic ductal adenocarcinoma cells and recruitment of TAMs, and neutralizing anti-CCL2 antibody selectively inhibits radiotherapy-dependent recruitment of TAMs and delays tumor growth in combination with radiotherapy [54].

Myeloid-derived suppressor cells (MDSCs) also foster cancer cell activity by suppression of T lymphocytes and natural killer cells, while simultaneously they can recruit regulatory T lymphocytes to further promote immunosuppression [55]. Deletion of CCL2 blocked progression from dysplasia to adenocarcinoma and reduced the number of colonic MDSCs in a spontaneous mouse model of colitis-associated colorectal cancer. CCL2 induces MDSC accumulation in evolving colonic tumors and enhances polymorphonuclear-MDSC immunosuppressive features, resulting in T lymphocyte suppression by polymorphonuclear-MDSCs in a STAT3-mediated manner [56]. In a mouse liver cancer model, fibroblast activation protein (FAP)–STAT3–CCL2 signaling in cancer-associated fibroblasts (CAFs) promoted tumor growth by enhancing MDSC mobilization and FAP-mediated tumor promotion, and MDSC mobilization in CAF was abolished in CCR2-deficient mice [57].

CAFs are stromal components and play an important role in tumor progression by regulating the tumor microenvironment and primarily releasing proteolytic enzymes, growth factors, and cytokines [58,59]. Pancreatic ductal adenocarcinoma cells educate fibroblasts through the secretion of CCL2 and IL-8, leading to CAFs and similar metastasis-related fibroblasts. CAFs and metastasis-related fibroblasts promoted angiogenesis and tumor progression by interacting with cancer cells [60]. Moreover, CCL2 involves tumor cell extravasation through the endothelium. Cancer cell-derived CCL2 activates vascular endothelial cells to increase vascular permeability and increases cancer cell extravasation and metastasis in in vivo colon cancer model. CCL2-induced vascular permeability and metastasis are dependent on Janus kinase2 (JAK2)–STAT5 and p38 Mitogen-activated Protein Kinase (MAPK) signaling [61]. In lung cancer cells, endothelial CCR2 expression is reported to be an important factor for cancer cell extravasation and pulmonary metastasis as well [23].

## 3. The CCL2–CCR2 Axis as a Biomarker

Consistent with the role of CCL2 in cancer progression, serum CCL2 levels and CCL2 expression levels in tumor tissue have been reported as potential biomarkers in a variety of cancer patients (Table 1).

### 3.1. Prostate Cancer

The usefulness of serum CCL2 in prostate cancer patients was supported by a study using the serum samples of 255 and 124 men with and without prostate cancer, respectively, which reported that serum CCL2 levels in men with prostate cancer were significantly higher than those without. In addition, among the 255 patients with prostate cancer, those with serum CCL2 level ≥ 320 pg/mL had significantly poorer overall survival and prostate cancer-specific survival, higher TNM stages, and worse histological grade than those with serum CCL2 level < 320 pg/mL, indicating the potential of serum CCL2 level as a prognostic biomarker as well [62]. The outcome of prostate cancer patients with CCL2-positive tissues was significantly worse with lower survival time than those patients with CCL2-negative tissues [28].

### 3.2. Colorectal Cancer

When the preoperative serum CCL2 concentration of 45 colorectal cancer patients was measured, the 5-year survival rate was significantly better in the group with low preoperative CCL2 level than in the group with high preoperative CCL2 level [63]. In a study examining CCL2 expression in 87 hepatectomy specimens of colorectal cancer liver metastases, CCL2 levels were associated with cancer progression and were prognostic markers of survival after hepatectomy for colorectal liver metastases [64]. As a result of examining the expression level of CCL2 from 245 colorectal cancer samples, the high expression level of CCL2 in the primary lesion was a significant marker for predicting liver metastasis [65].

### 3.3. Hepatocellular Carcinoma (HCC)

Serum CCL2 was significantly higher in HCC patients in a study that measured CCL2 levels from serum samples from 98 resectable HCCs, 101 patients with chronic hepatitis B, and 100 asymptomatic hepatitis B and C virus carriers. In addition, the serum CCL2 also correlated with the disease stage of HCC and the combination of alpha-fetoprotein and CCL2 has significantly superior discriminative ability than alpha-fetoprotein alone [66]. In examining resected specimens of 57 HCC patients, the CCL2 gene was one of the predictive markers for the survival of HCC, especially in early-stage disease [67].

### 3.4. Breast Cancer

Serum CCL2 levels in 135 breast cancer patients were measured and elevated and serum CCL2 levels were significantly correlated with advanced cancer stage and lymph node metastasis [68]. Immunohistochemistry to examine the frequency of TAMs and CCL2-expressing cells from paraffin blocks of 137 breast cancer patients found that high frequency of CCL2 positive cancer cells and CD14+ TAMs were significant risk factors for cancer recurrence [69]. Analysis of 427 breast cancer specimens suggests that stromal CCL2 is associated with reduced recurrence-free survival in patients with basal-like breast cancer and may be a predictor of prognosis [70]. Analysis of 151 breast cancer samples showed that high expression of CCL2 and vascular endothelial growth factor were a significant indicator of early recurrence of breast cancer [71]. Immunohistochemical analysis of CCL2 expression from 27 breast invasive ductal carcinoma specimens showed that CCL2 expression in tumor parenchyma significantly correlated with the histological grade of ductal infiltrative breast cancer [72].

### 3.5. Pancreatic Cancer

A study of 212 pancreatic cancer patients found that CCL2 serum levels in pancreatic cancer patients were significantly higher than in normal healthy subjects [73]. In a study examining serum CCL2 levels in 68 patients with pancreatic cancer, a high level of serum CCL2 was strongly associated with poor survival and prognosis [74]. An analysis of 483 pancreatic cancer patients showed that patients with pancreatic cancer with high CCL2 expression and low CD8+ T lymphocyte infiltration had significantly reduced survival [75]. CCL2 and IL-8 cytokine levels were found to be associated with poorer survival after pancreatic resection in a study analyzing the sera of 85 pancreatic cancer patients and 23 patients with benign pancreatic tumors [76]. CCL2 was found to be a ubiquitously expressed gene, including expression in adipose tissue, skeletal muscle, and pancreatic cancer cells, and was associated with cachexia in pancreatic cancer patients [77].

### 3.6. Malignant Lymphoma

CCL2 and CCR2 expression was analyzed by immunohistochemical staining and its correlations with clinicopathologic features and prognosis were evaluated in 221 patients with diffuse large B-cell lymphoma, and high expression of CCL2 or CCR2 was correlated with clinicopathological characteristics and indicated significantly poorer overall survival and progression-free survival [78]. In a study in 81 lymphoma patients, including 44 non-Hodgkin’s lymphoma (NHL), 37 Hodgkin’s lymphoma patients, and 20 healthy subjects as a control group, high serum levels of inflammatory markers, including CCL2, correlate with severity and low benefit from treatment [79]. In a study using samples of 20 NHL patients and 8 reactive tonsils, the level of CCL2 cDNA in patients with aggressive NHL was higher than in indolent tumors [80].

### 3.7. Gastric Cancer

As a result of examining the peripheral blood of 60 patients with gastric cancer and 20 healthy subjects, preoperative serum levels of CCL2 in patients with gastric cancer were significantly higher than those in healthy controls. In addition, preoperative serum CCL2 was significantly correlated with lymph node metastasis [81]. A study of serum CCL2 in 78 patients with gastric cancer and 30 healthy controls showed that patients with gastric cancer with elevated serum CCL-2 levels were significantly less responsive to chemotherapy [82]. Measuring chemokine levels from the plasma of 66 gastric cancer patients and 11 healthy controls revealed CCL2 levels correlated with advanced gastric cancer stage [83]. In immunohistochemistry of 68 patients with gastric cancer, high CCL2 expression in tumor tissue showed significantly poor prognosis [84]. Similarly, as a result of immunohistochemical examination from clinical specimens of 178 gastric cancer patients, CCL2 and Snail were independent prognostic factors of gastric cancer [85]. Immunohistochemical staining of CCL2 expression in tumor tissue in 414 gastric cancer patients who underwent gastrectomy showed that the increased expression level of CCL2 was an independent prognostic factor for overall survival in patients with gastric cancer [86].

### 3.8. Ovarian Cancer

In a retrospective study including 86 ovarian cancer patients, 67 benign ovarian cysts, and 42 healthy women, ovarian cancer patients had significantly higher serum CCL2 levels, and serum CCL2 levels were statistically significantly correlated with histological malignancy [87]. In a study investigating CCL2 gene expression using tumor specimens from 37 ovarian cancer patients, CCL2 mRNA expression was correlated with objective complete response, chemosensitivity, and progression-free survival [88].

### 3.9. Lung Cancer and Malignant Pleural Mesothelioma

Comparing 142 lung cancer patients with 141 non-lung cancer subjects, CCL2 was significantly elevated in lung cancer patients [89]. Western blotting results from tissue specimens in 719 patients with lung cancer revealed that patients with high CCL2 expression had significantly worse overall survival and progression-free survival [90]. In a study of 50 patients with malignant pleural mesothelioma who may have been exposed to asbestos, 356 subjects who may have been exposed to asbestos but had no disease, and 41 healthy volunteers with no history of asbestos exposure, serum CCL2 levels were significantly elevated in patients with advanced malignant pleural mesothelioma [91].

### 3.10. Bladder and Kidney Cancers

Examining CCL2 levels in urine samples from 60 bladder cancer patients and 20 control healthy subjects revealed CCL2 levels in the urine of patients with bladder cancer correlated significantly with TNM stage and cancer grade [92]. Immunohistochemical analysis of 114 kidney surgical specimens showed that upregulation of CCL2 expression correlated with macrophage infiltration, clinical stage, and overall survival [93]. CCL2 and CCR2 were identified as independent risk factors for overall survival and recurrence-free survival after surgical treatment from a sample of 268 patients with non-metastatic kidney cancer [94].

### 3.11. Head and Neck Cancers

Examination of serum CCL2 and TNF-α in 297 patients with pretreatment laryngeal cancer showed that CCL2 and TNF-α levels were independent predictors of overall survival and distant metastasis-free survival [95]. Immunohistochemical staining of tissue specimens of 115 patients with papillary thyroid cancer showed that CCL2 expression levels were an independent predictor of recurrence [96]. CCL2 levels in cerebrospinal fluid samples from patients with malignant glioma were significantly higher than those from patients with benign glioma or healthy subjects [97].

## 4. The Development of the CCL2–CCR2 Axis-Targeted Agents

The effects of the orally administered CCR2 inhibitor PF-04136309 in combination with leucovorin (folinic acid), fluorouracil, irinotecan, and oxaliplatin (FOLFIRINOX) in patients with treatment-naive locally advanced pancreatic ductal adenocarcinoma were assessed in a phase Ib study; ultimately, 16 (49%) of 33 patients receiving FOLFIRINOX plus PF-04136309 achieved an objective tumor response, with local tumor control achieved in 32 (97%) patients [98]. However, in another phase Ib study, assessing PF-04136309 in combination with nab-paclitaxel and gemcitabine in 21 patients with previously untreated metastatic pancreatic ductal adenocarcinoma, the objective response rate with this treatment was only 23.8% [99]. The clinical utility of carlumab, a human antibody with high affinity and specificity for CCL2, was investigated in a phase I study of 44 patients with solid malignancies. In this study, patients with ovarian and prostate cancer achieved more than 50% CA125 and prostate-specific antigen (PSA) reduction and retained stable disease for 10.5 and 5 months, respectively [100]. The clinical benefit of carlumab was also investigated in 46 patients with metastatic castration-resistant prostate cancer previously treated with docetaxel in a phase II, open-label study. However, carlumab alone did not block the CCL2–CCR2 axis or show antitumor activity [101]. MLN1202, a humanized, neutralizing anti-CCR2 antibody was investigated in a phase II clinical trial for the treatment of 44 patients with bone metastasis, regardless of the primary site. Forty-one out of 43 eligible patients completed this study, with 7% having serious adverse events. The concentration in urine of N-telopeptide, a biomarker to measure bone turnover rates, decreased in 14% of the patients, suggesting a positive effect of the antibody in these patients [102]. A multicenter clinical trial was conducted in patients with locally advanced or metastatic unresectable pancreatic cancer who received the CCR2-specific antagonist CCX872 in combination with FOLFIRINOX [103]. Fifty patients with pancreatic cancer were treated and the 18-month overall survival rate was 29%. The previously reported 18-month overall survival rate for the FOLFIRINOX single-agent regimen was 18.6%, indicating the better treatment benefit in the combination therapy than single agents [104]. Various clinical trials targeting the CCL2–CCR2 axis are currently underway as shown in Table 2 and the results are awaited. Not only clinical trials to examine the effect of CCR2 inhibitor but also investigations to narrow down the patients who can be expected the effect of the CCL2–CCR2 axis targeted agents by confirming the expression of CCR2 with imaging tests are conducted.

Losartan, a type I angiotensin II receptor antagonist, is reported to have the potential to act as direct CCR2 antagonists. Losartan and its primary metabolite, EXP-3174, inhibit CCL2-mediated monocyte recruitment to the metastatic site through the inhibition of CCL2-induced Erk1/2 activation and significantly reduce the metastatic burden in breast and colon cancer models [105]. Similarly, type I angiotensin II receptors were reported to regulate CCL2 in prostate cancer and pancreatic cancer cells, suggesting that losartan, and potentially other type I angiotensin II receptor blockers, could be repurposed for use in cancer immunotherapy [106,107].

Preclinical studies using the mouse model were also reported. Inhibition of CCL2 improved radiation resistance using mice transplanted with pancreatic duct adenocarcinoma cell lines [54]. In a preclinical study using mouse transplanted with a cabazitaxel-resistant prostate cancer cell line, inhibition of the CCL2–CCR2 axis released cabazitaxel-resistance and suppressed tumor growth in mice treated concurrently with cabazitaxel and CCR2 antagonist [30]. Administration of CCR2 inhibitor to liver metastasis model mice for colorectal cancer improved responsiveness to chemotherapy and prolonged overall survival [6]. Interestingly, in a mouse model of metastatic breast cancer, administration of anti-CCL2 antibody suppressed metastasis, while interruption of CCL2 inhibition caused metastatic overshoot and accelerated death [108]. These results may confuse the story of mechanisms in the CCL2–CCR2 axis contributing to cancer progression.

## 5. Discussion

Although many studies have proven the key roles of the CCL2–CCR2 axis in tumor progression in a variety of cancers, there are no clinically available drugs that can modulate the CCL2–CCR2 axis as anticancer agents so far. As described earlier, a high serum CCL2 level may reflect the activity of cancer cells because CCL2 is produced by them; however, some studies have reported conflicting results that a low serum CCL2 level contributed to a worse prognosis in cancer patients [109]. This discrepancy among clinical data suggests that there may be unidentified mechanisms of the CCL2–CCR2 axis with regard to the inhibition of cancer cell activation, and the dynamics of CCL2 in sera and the tumor microenvironment may be different among various cancers. Moreover, the application of agents for the blockade of the CCL2–CCR2 axis may be narrowed down to obtain better efficacy. As cancer cells or associated cells in the tumor microenvironment must express CCR2 at a high level, immunohistochemistry analysis of cancer tissue from patients may be needed and patients may have to be selected properly, in addition to previous confirmation of CCR2 expression in multiple cancer cell lines. The CCL2–CCR2 axis may be more important in cancers with high immunogenicity than those with low immunogenicity and that can hardly mobilize immune cells into the tumor microenvironment. CCL2 activates TAMs and MDSCs that can activate regulatory T lymphocytes. Regulatory T lymphocytes can inhibit the activity of cytotoxic T lymphocytes that play a key role in the immune system to eliminate cancer cells in the tumor microenvironment [55]. According to this sequential mechanism, programmed death-ligand 1 or programmed cell death protein 1 inhibitors that impede immune tolerance between cytotoxic T lymphocytes and cancer cells may synergistically function with the blockade of the CCL2–CCR2 axis as a combination anticancer treatment. Interestingly, the therapeutic effects of combination treatment combining CCL2 antagonist and anti-programmed death-ligand 1 antibody in lung tumor-bearing mice decreased MDSC in the periphery and tumor through an enhanced CD4+ and CD8+ T lymphocyte infiltration and increased the survival time compared to single agent alone [110].

The performance of the drug itself in exerting its potential effect to suppress cancer cell activity is also important to consider. Measurement of free CCL2 concentration following carlumab administration revealed a rapid fall in free CCL2 as expected; however, post-treatment free CCL2 concentrations rapidly rebounded and quickly exceeded pretreatment serum concentrations. These unexpected results suggest that carlumab was ineffective at suppressing free CCL2 concentration for a clinically meaningful period of time. As no apparent increase in CCL2 production rate was observed when the dose was increased from 1 to 50 mg/kg in cynomolgus monkeys, dose-escalation might be needed to adequately and constitutively suppress free CCL2 concentration [101]. PF-04136309 showed that the level of CCR2-positive inflammatory monocytes decreased in the peripheral blood but did not accumulate in the bone marrow [99]. The chemical modification of these agents may improve the anticancer effects.

The CCL2–CCR2 axis functions physiologically in various organs such as the lungs and digestive tract where a variety of immune cells accumulate. Blockade of the CCL2–CCR2 axis potentially causes unexpected effects in such organs. Although it is unclear whether each adverse event attributes to the blockade of the CCL2–CCR2 axis because PF-04136309 was used in combination with cytotoxic agents in a single-arm study, pulmonary toxicity and diarrhea as well as myelosuppression were reported as severe adverse events in clinical trials [99]. Although the incidence of treatment-related severe adverse events was not so high, grade 4 gastrointestinal perforation and grade 5 multi-organ failure were also reported in carlumab study for prostate cancer patients even any other anti-cancer agents were concurrently started [98,99,101]. In case an agent designed for the blockade of the CCL2–CCR2 axis is used in combination therapy, strict attention should be paid to the choice of agents being used together not to amplify treatment-related adverse events. However, the adverse events of blocking the CCL2-CCR2 axis are still not fully investigated, and further data should be accumulated in future clinical trials. As previously mentioned, losartan, and potentially other type I angiotensin II receptor blockers, might be repurposed for use in cancer immunotherapy to prevent severe adverse events; however, the clinically effective dose as anticancer agents should be investigated [106,107]. A recent study reported that the coffee compound kahweol acetate and cafestol can reduce not only CCL2 secretion but also CCR2 expression on prostate cancer cells [111]. These food-derived agents may be potential candidates for therapy in the future, especially as part of a combination therapy approach for the treatment of cancers with high immunogenicity.

In summary, to achieve sufficient efficacy of CCL2–CCR2-targeted therapy for cancer patients, it is not sufficient to target cancer cells or immune cells alone and it is necessary to fully elucidate the physiological functions of the CCL2–CCR2 axis against a variety of normal cells expressing CCR2.

## Figures and Tables

**Table 1:** Role of CCL2–CCR2 axis as a biomarker in various cancers.

Cancer Types	Target	Size	Specimens	Biomarker	Variables	References
Prostate cancer	Men with and without prostate cancer	379	Blood tissue	High serum CCL2 levels	Prostate cancer	[62]
	Prostate cancer patients	255		Serum CCL2 level ≥ 320 pg/mL	Poor OS, Poor PCSS, Advanced cancer stage, Poor histological grade	
Prostate cancer	Men with and without prostate cancer	55	Prostate tissue	CCL2 positive in tissue	Prostate cancer	[28]
	Prostate cancer patients	35	Primary tumor tissue		Poor OS	
Colorectal cancer	Preoperative colorectal cancer patients	45	Blood tissue	Preoperative serum CCL2 levels	Poor 5-year survival rate	[63]
Colorectal cancer	Colorectal cancer patients with liver metastases	87	Hepatectomy tissue	High expression of CCL2 levels	Poor survival after hepatectomy for colorectal liver metastases	[64]
Colorectal cancer	Colorectal cancer patients	245	Primary tumor tissue	High expression of CCL2 levels	Liver metastasis	[65]
HCC	Resectable HCC patients, Chronic hepatitis B patients, Asymptomatic hepatitis B and C virus carriers	299	Blood tissue	High serum CCL2 levels	HCC	[66]
				High serum alpha-fetoprotein and CCL2 levels	Disease stage of HCC	
HCC	HCC patients	57	Primary tumor tissue	High expression of CCL2 gene	Poor survival of HCC in early-stage disease	[67]
Breast cancer	Breast cancer patients	135	Blood tissue	High serum CCL2 levels	Advanced cancer stage, Lymph node metastasis	[68]
Breast cancer	Breast cancer patients	137	Primary tumor tissue	High frequency of CCL2 positive cancer cells and CD14+ TAMs	Cancer recurrence	[69]
Breast cancer	Breast cancer patients	427	Primary tumor tissue	Stromal CCL2	Poor RFS with basal-like breast cancer	[70]
Breast cancer	Breast cancer patients	151	Primary tumor tissue	High expression of CCL2 and VEGF	Cancer recurrence	[71]
Breast cancer	Breast invasive ductal carcinoma patients	27	Primary tumor tissue	High expression of CCL2	Poor histological grade	[72]
Pancreatic cancer	Suspected pancreatic neoplasm patients	212	Blood tissue	High serum CCL2 levels	Pancreatic cancer	[73]
Pancreatic cancer	Pancreatic cancer patients	68	Blood tissue	High serum CCL2 levels	Poor survival and prognosis	[74]
Pancreatic cancer	Pancreatic cancer patients	483	Primary tumor tissue	High CCL2 expression, Low CD8+ T lymphocyte infiltration	Poor survival	[75]
Pancreatic cancer	Pancreatic cancer patients, Patients with benign pancreatic tumors	108	Blood tissue	High serum CCL2 and IL-8 cytokine levels	Poor survival after pancreatic resection	[76]
Pancreatic cancer	Pancreatic cancer patients	70	Primary tumor tissue	High expression of CCL2	Cachexia	[77]
Malignant lymphoma	Diffuse large B-cell lymphoma patients	221	Primary tumor tissue	High expression of CCL2 and CCR2	Poor OS, Poor PFS, Poor clinicopathological characteristics	[78]
Malignant lymphoma	Non-Hodgkin’s lymphoma patients, Hodgkin’s lymphoma patients, Healthy subjects	81	Blood tissue	High serum CCL2 levels	Severity, Low benefit from treatment	[79]
Malignant lymphoma	Non-Hodgkin’s lymphoma patients, Reactive tonsils	28	Primary tumor tissue	High expression of CCL2	Poor histological grade	[80]
Gastric cancer	Gastric cancer patients, Healthy subjects	80	Blood tissue	High serum CCL2 levels	Gastric cancer	[81]
				Preoperative serum CCL2 levels	Lymph node metastasis	
Gastric cancer	Gastric cancer patients, Healthy subjects	108	Blood tissue	High serum CCL2 levels	Poor responsive to chemotherapy	[82]
Gastric cancer	Gastric cancer patients, Healthy subjects	77	Blood tissue	High serum CCL2 levels	Advanced gastric cancer stage	[83]
Gastric cancer	Gastric cancer patients	68	Primary tumor tissue	High expression of CCL2	Poor prognosis	[84]
Gastric cancer	Gastric cancer patients	178	Primary tumor tissue	High expression of CCL2 and Snail	Poor prognosis	[85]
Gastric cancer	Gastric cancer patients	414	Primary tumor tissue	High expression of CCL2	Poor OS	[86]
Ovarian cancer	Ovarian cancer patients, Benign ovarian cyst patients, Healthy women	195	Blood tissue	High serum CCL2 levels	Ovarian cancer, Poor histological malignancy	[87]
Ovarian cancer	Ovarian cancer patients	37	Primary tumor tissue	High expression of CCL2 mRNA	Objective complete response, Poor chemosensitivity, Poor PFS	[88]
Lung cancer	Lung cancer patients, Non-lung cancer subjects	283	Blood tissue	High serum CCL2 levels	Lung cancer	[89]
Lung cancer	Lung cancer patients	719	Primary tumor tissue	High expression of CCL2	Poor OS, Poor PFS	[90]
Malignant pleural mesothelioma	Malignant pleural mesothelioma patients, Healthy subjects	447	Blood tissue	High serum CCL2 levels	Advanced malignant pleural mesothelioma	[91]
Bladder cancer	Bladder cancer patients	80	Urine	High CCL2 levels in the urine	Advanced cancer stage, Poor histological grade	[92]
Kidney cancer	Kidney cancer patients	114	Primary tumor tissue	High expression of CCL2	Advanced clinical stage, Poor OS	[93]
Kidney cancer	Non-metastatic kidney cancer patients	268	Primary tumor tissue	High expression of CCL2 and CCR2	OS after surgical treatment, RFS after surgical treatment	[94]
Laryngeal cancer	Laryngeal cancer patients	297	Blood tissue	High serum CCL2 and TNF-α levels	Poor OS, Poor distant metastasis-free survival	[95]
Thyroid cancer	Papillary thyroid cancer patients	115	Primary tumor tissue	High expression of CCL2	Cancer recurrence	[96]
Malignant glioma	Malignant glioma patients, Benign glioma patients, Patients with nontumor disorders of the central nervous system	35	Cerebrospinal fluid	High CCL2 levels	Malignant glioma	[97]

CCL2, C-C Motif Ligand 2; CCR2, C-C Chemokine receptor 2; OS, overall survival; PCSS, prostate cancer-specific survival; HCC, hepatocellular carcinoma; TAM, tumor-associated macrophage; RFS, recurrence-free survival; VEGF, vascular endothelial growth factor.

**Table 2:** Ongoing clinical trials of CCR2 inhibitors and CCR2 imaging.

Trial ID	Intervention	Target	Randomize	Patient Size
For treatment				
NCT03496662	Arm A: BMS-813160 ^1^/Nivolumab/Gemcitabine/Nabpaclitaxe Arm B: Gemcitabine/Nab-paclitaxel Arm C: BMS-813160/Nivolumab/Gemcitabine/Nabpaclitaxel (dose expansion)	Locally advanced pancreatic ductal adenocarcinoma	No	53
NCT03767582	Phase I Arm A: BMS-813160/Nivolumab/GVAX ^2^ Phase II Arm B: BMS-813160/Nivolumab Arm C: BMS-813160/Nivolumab/GVAX	Locally advanced pancreatic ductal adenocarcinoma	Yes	30
NCT04123379	Non-small cell lung cancer Arm A: Nivolumab/BMS-813160 Arm B: Nivolumab/BMS-986253 ^3^ Hepatocellular carcinoma Arm C: Nivolumab Arm D: Nivolumab/BMS-813160 Arm E: Nivolumab/BMS-986253	Non-small cell lung cancer Hepatocellular carcinoma	Yes	50
NCT03184870	Arm A: BMS-813160/5-FU/Leucovorin/Irinotecan Arm B: BMS-813160/Nab-paclitaxel/Gemcitabine Arm C: BMS-813160/Nivolumab Arm D: BMS813160/Nabpaclitaxel/Gemcitabine/Nivolumab Arm E: 5-FU/Leucovorin/Irinotecan Arm F: Nab-paclitaxel/Gemcitabine Arm G: BMS-813160	Colorectal cancer Pancreatic cancer	No	348
NCT02996110	Arm A: Nivolumab/Ipilimumab Arm B: Nivolumab/Relatlimab Arm C: Nivolumab/BMS-986205 Arm D: Nivolumab/BMS-813160	Advanced renal cell carcinoma	Yes	200
For diagnosis				
NCT03851237	Arm 1A: Receive treatment with upfront surgery such as Whipple procedure/64Cu-DOTA-ECL1i-PET-CT imaging ^4^ Arm 1B: Standard of care chemotherapy/64CuDOTA-ECL1i-PET-CT imaging Arm 2: CCR2-targeted therapy/64Cu-DOTA-ECL1i-PET-CT imaging	Pancreatic ductal adenocarcinoma	No	75
NCT04537403	Arm 1A: Normal volunteers and patients with Carotid and Femoral Atherosclerosis who will be having surgery/64Cu-DOTA-ECL1i-PET-CT imaging Arm 1B: Patients with Carotid and Femoral Atherosclerosis who will be managed medically and not having surgery/64Cu-DOTA-ECL1i-PET-CT imaging	Carotid atherosclerosis	No	100

^1^ CCR2/5-inhibitor; ^2^ granulocyte-macrophage colony-stimulating factor-secreting allogeneic tumor cells; ^3^ human monoclonal antibody that binds to and inhibits IL-8; ^4^ Imaging CCR2 Receptors; 5-FU, 5fluorouracil; 64Cu-DOTA-ECL1i-PET-CT imaging, Imaging CCR2 Receptors.

## Abbreviations

Akt	Protein kinase B
CAF	Cancer-associated Fibroblast
CCL2	C-C motif ligand 2
CCR2	C-C Chemokine Receptor 2
EMT	Epithelial-Mesenchymal Transition
FAP	Fibroblast Activation Protein
FOLFIRINOX	leucovorin, fluorouracil, irinotecan, and oxaliplatin
HCC	Hepatocellular Carcinoma
IL	Interleukin
JAK2	Janus kinase2
MAPK	Mitogen-activated Protein Kinase
MDSC	Myeloid-Derived Immune Suppressor Cell
MMP	Matrix Metalloproteinase
NHL	Non-Hodgkin’s Lymphoma
PIPKIγ	Phosphatidylinositol phosphate kinase gamma
PI3K	Phosphatidylinositol 3-kinase
PSA	Prostate-specific antigen
STAT3	Signal Transducers and Activator of Transcription 3
TAM	Tumor-Associated Macrophage
